# Effects of a single aerobic exercise on perfused boundary region and microvascular perfusion: a field study

**DOI:** 10.1007/s10877-021-00660-w

**Published:** 2021-02-03

**Authors:** Alexander Fuchs, Tobias Neumann, Hendrik Drinhaus, Anika Herrmann, Hans Vink, Thorsten Annecke

**Affiliations:** 1 Faculty of Medicine and University Hospital of Cologne, Department of Anaesthesiology and Intensive Care Medicine, University of Cologne, Cologne, Germany; 2 Department of Physiology, Maastricht University Medical Center, Maastricht, The Netherlands; 3 Department of Anaesthesiology and Intensive Care Medicine, University of Witten/ Herdecke, Kliniken der Stadt Köln gGmbH, Cologne, Germany

**Keywords:** Endothelial surface layer, Glycocalyx, Perfused boundary region, Capillary density, Intravital microscopy, Sidestream darkfield imaging

## Abstract

The endothelium and the glycocalyx play a pivotal role in regulating microvascular function and perfusion in health and critical illness. It is unknown today, whether aerobic exercise immediately affects dimensions of the endothelial surface layer (ESL) in relation to microvascular perfusion as a physiologic adaption to increased nutritional demands. This monocentric observational study was designed to determine real-time ESL and perfusion measurements of the sublingual microcirculation using sidestream dark field imaging performed in 14 healthy subjects before and after completing a 10 km trial running distance. A novel image acquisition and analysis software automatically analysed the perfused boundary region (PBR), an inverse parameter for red blood cell (RBC) penetration of the ESL, in vessels between 5 and 25 µm diameter. Microvascular perfusion was assessed by calculating RBC filling percentage. There was no significant immediate effect of exercise on PBR and RBC filling percentage. Linear regression analysis revealed a distinct association between change of PBR and change of RBC filling percentage (regression coefficient β: − 0.026; 95% confidence interval − 0.043 to − 0.009; p = 0.006). A single aerobic exercise did not induce a change of PBR or RBC filling percentage. The endothelium of the microvasculature facilitates efficient perfusion in vessels reacting with an increased endothelial surface layer.

## Introduction

Microvascular perfusion is adapted to local needs by fine-tuned adaptive mechanism. In this context, the endothelial glycocalyx and the endothelium play a pivotal role in regulating microvascular function and perfusion. The glycocalyx is composed of proteoglycans and glycosaminoglycans on its luminal site. It is part of the endothelial surface layer (ESL) that contains associated soluble plasma proteins and immobilised plasma. The ESL is involved in many physiological functions such as mechanotransduction, haemostasis, signalling, and blood cell–vessel wall interactions [1, 2]. Given these essential functions, ESL degradation will lead to formation of tissue edema, loss of nutritional blood flow and leucocyte adhesion in states of critical illness such as sepsis, ischaemia–reperfusion-injury and trauma [2]. Experimental models of ischaemia/reperfusion and hypoxia/reoxygenation showed that myocardial hypoxia and ischaemia induce an early ESL degradation [3], with purin signalling and perivascular mast cell-derived heparanase being a possible contributing factor [4]. Increased blood levels of shed ESL components such as syndecan-1, hyaluronan and heparan sulphate can be detected in successful cardiopulmonary resuscitation after cardiac arrest, trauma and sepsis and may serve as an early predictor of adverse clinical outcome [5–7]. Soluble components of the endothelial glycocalyx may act as damage associated molecular patterns (DAMPs), further augmenting inflammation and tissue damage. Therefore, eliminating these substances from the circulation is under evaluation as a therapeutic intervention [8].

In order to visualise and calculate ESL dimensions, novel non-invasive techniques are available to study the microcirculation in regions that are easily accessible. Recently, clinical studies using intravital microscopy of the sublingual microvasculature have been conducted to assess the ESL in various medical conditions and critical care states [9–14]. These measurements were performed in patients and controls either in-hospital or in a laboratory setting.

However, rapid changes of ESL dimensions may occur. To this date, it is unknown whether aerobic exercise immediately affects dimensions of the endothelial surface layer and hereby adapts local tissue perfusion. The current study aimed to investigate the microvascular function of healthy adults using the above mentioned non-invasive techniques before and after running a 10 km trial distance. We hypothesised that aerobic exercise will induce a physiological change of ESL dimensions in order to facilitate efficient perfusion. Furthermore, we tested the feasibility of post-exercise on-site measurements to elucidate alternative scenarios for intravital microscopy other than an in-hospital or a laboratory setting.

## Methods

### Study design, setting and subjects

This was a monocentric, observational field study to obtain the effect of aerobic exercise on real-time ESL and perfusion measurements of the sublingual microcirculation in 14 healthy adults. Measurements were performed on site before and after running a 10 km distance trail race consisting of 4 rounds with a total difference in altitude of 72 m in a public park. The 10 km route had been measured and predefined beforehand. Transponder timing of every participant had been established. A generator was used to meet the power demand of the videomicroscope and ensure the performance of the connected notebook. Upon enrolment, every participant was asked to fill in a questionnaire about health and long-term medication. Standard vital parameters were obtained before and after the endurance run.

The primary endpoint was the perfused boundary region (PBR) of vessels between 5 and 25 µm diameter as an inverse parameter for ESL dimensions. PBR is the depth of lateral erythrocyte penetration into ESL. Its calculation has been described elsewhere [15]. Secondary endpoints were PBR of the sublingual microvasculature graded according to vessel size, red blood cell (RBC) filling percentage as a parameter for microvascular perfusion and change of the mentioned parameters between post-exercise and baseline. Valid and total capillary density as well as the ratio of valid to total capillary density served as an additional marker for microvascular perfusion and capillary recruitment, respectively.

The study was conducted in accordance with the Declaration of Helsinki and its protocol has been approved by the local Ethics Committee of the University of Cologne (AZ 18–162). All participants gave written informed consent prior to any study-related procedures.

### Assessment of ESL dimensions and microvascular perfusion

Participants underwent non-invasive imaging of the sublingual microvasculature via intravital microscopy using a handheld sidestream darkfield videomicroscope (Capiscope HVCS Handheld Video Capillaroscopy System, KK Research Technology, Devon, UK). Properties of the endothelial surface layer and quality of microvascular perfusion were analysed using GlycoCheck™ software (Microvascular Health Solutions, Orem, Utah, USA). During acquisition the software provides feedback regarding stability and focus and adjusts light intensity. This ensures that only adequate images are being recorded for automatic analysis and thus limits inter-observer variability.

Each participant underwent two baseline und two post-exercise measurements from which the mean PBR was calculated to provide a single PBR value per subject per measurement. The process of image acquisition and reproducibility of imaging and analysis via intravital microscopy and the above mentioned software have been described elsewhere [12–14].

The perfused boundary region (PBR) is an inverse parameter for RBC penetration of the endothelial surface layer, and hence, its thickness. RBC filling percentage is the percentage of time in which valid vessel segments have RBCs present and is an estimate for microvascular perfusion [15]. RBC filling percentage is determined in individual vascular segments and is defined as the fractional longitudinal RBC content without assessing haemodynamics. The presence of RBCs along the length of a vascular segment is determined by measuring radial intensity profiles at 21 positions in 40 sequential video frames, resulting in 840 potential RBC detections. RBC filling percentage is the number of positive RBC detections relative to the maximal number of 840. The change of PBR and RBC filling percentage was calculated by subtracting the baseline from the post-exercise value. The detailed definition of valid and total capillary density is provided elsewhere [16].

### Statistical analysis

As this was a pilot study and a field test for the method itself, we did not conduct a sample size estimation. Analysis of within-group differences was carried out using Wilcoxon test. Normally distributed variables are presented as mean ± standard deviation; for non-normally distributed data the median and interquartile range are given. Spearman’s correlation was used to test independence between variables. Linear regression analysis was performed to investigate the associations of the change of PBR and change of RBC filling percentage. The significance threshold was set at 0.05 (two-tailed). Calculations were performed with SPSS Statistics v25 (IBM Corp., Armonk, NY, USA). Data visualisation was conducted in RStudio 1.2.5019 for R [17] with the package ‘gplot2’ [18].

## Results

### Study population

Participants’ clinical and demographic characteristics pre- and post-exercise are displayed in Table 1. Fourteen young to middle-aged, normal weight, normotensive adults were included, a third being female. Ten kilometre trial running time ranged from 48 to 59 min. Post-exercise there was an increase of heart rate accompanied by a slight decrease in systolic blood pressure and peripheral oxygen saturation.

**Table 1:** Participant characteristics and pre- and post-exercise vital parameters

	n = 14
Age (years)	36 ± 8
Gender (female)	5 (35.7)
Height (m)	1.82 [1.74–1.88]
Weight (kg)	75 ± 12
BMI (kg/m^2^)	22.9 ± 3.0
Men	23.9 ± 3.2
Women	21.2 ± 1.5

	Baseline	Post-exercise	p
Heart rate (beats/min)	70 [65–77]	105 [95–118]	0.001
Systolic blood pressure (mmHg)	125 [120–140]	120 [110–130]	0.012
Diastolic blood pressure (mmHg)	80 [70–90]	80 [80–90]	0.217
SpO_2_ (%)	98 [98–98]	97 [95–97]	0.021
Ten kilometre running time (mm:ss)	53:26 ± 03:42		
	51:38 [51:11–57:48]		

*BMI* body mass index, *SpO*_*2*_ peripheral oxygen saturation

Values are given as mean ± standard deviation, frequency (percentage) median or median [1. quartile –3. quartile]

### ESL dimension and perfusion measurements

**Fig. 1:**
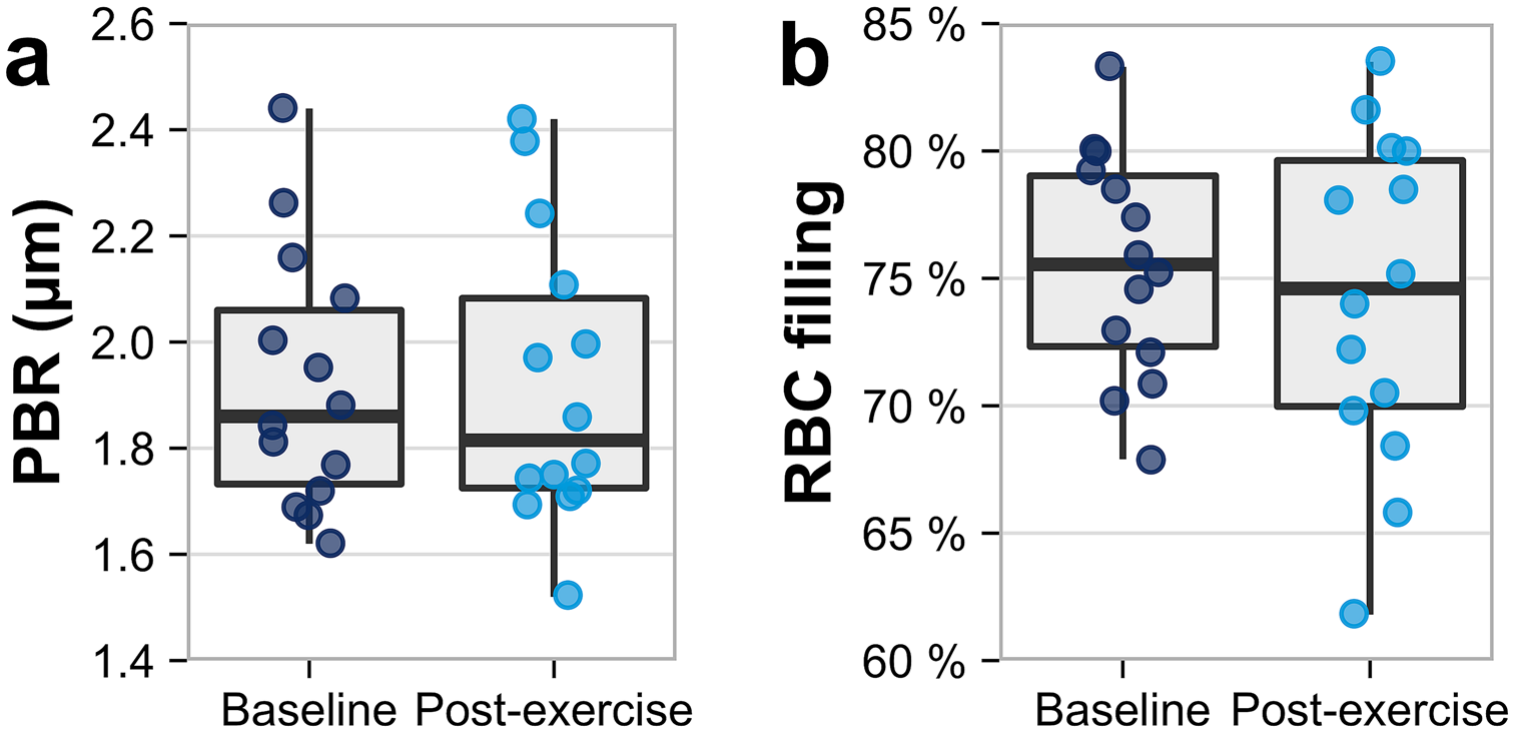
**a** PBR (µm) in vessels between 5 and 25 µm diameter at baseline and post-exercise as an inverse parameter for RBC penetration into the ESL. **b** RBC filling (%) at baseline and post-exercise. The whiskers extend from Q1 and Q3 to minimum and maximum of the data sets

The baseline PBR of vessels between 5 and 25 µm diameter was 1.86 [1.72–2.08] µm. Baseline RBC filling percentage was 75.6 [72.1–79.2]%. There was no significant immediate effect of aerobic exercise on PBR of 5 to 25 µm vessels (1.82 [1.72–2.11] µm, p = 0.975), RBC filling percentage (74.6 [69.8–80.0]%, p = 0.397) (Fig. 1) or differentiated PBR according to vessel size (i.e. 5 to 9, 10 to 19 and 20 to 25 µm) (data not shown).

The baseline valid capillary density was 453 [340–521] n/mm^2^ and the total capillary density 731 [614–823] n/mm^2^. There was no significant immediate effect on valid capillary density (457 [350–492] n/mm^2^, p = 0.660) or total capillary density post-exercise (693 [616–904] n/mm^2^, p = 0.950). The baseline ratio of valid to total capillary density was 57 [48–67]%. There was no significant immediate effect of aerobic exercise on the ratio of valid to total capillary density (60 [49–67]%, p = 0.975).

**Fig. 2:**
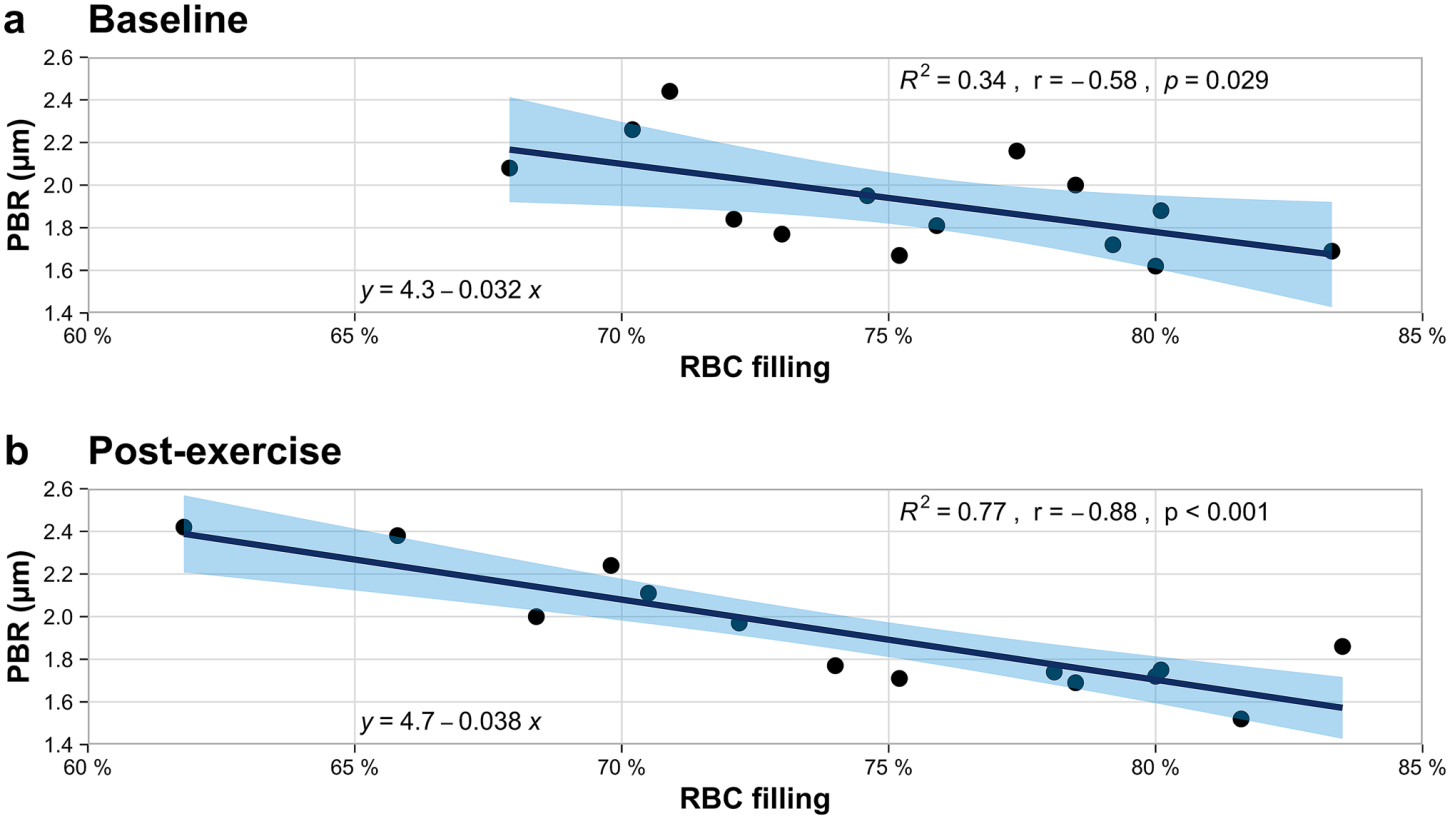
**a** Scatterplot of PBR (%) and RBC filling (%) at baseline. **b** Scatterplot of PBR (%) and RBC filling (%) post-exercise. Linear regression lines overlaid, coefficients of determination, Spearman’s correlation coefficients and p-values are given

**Fig. 3:**
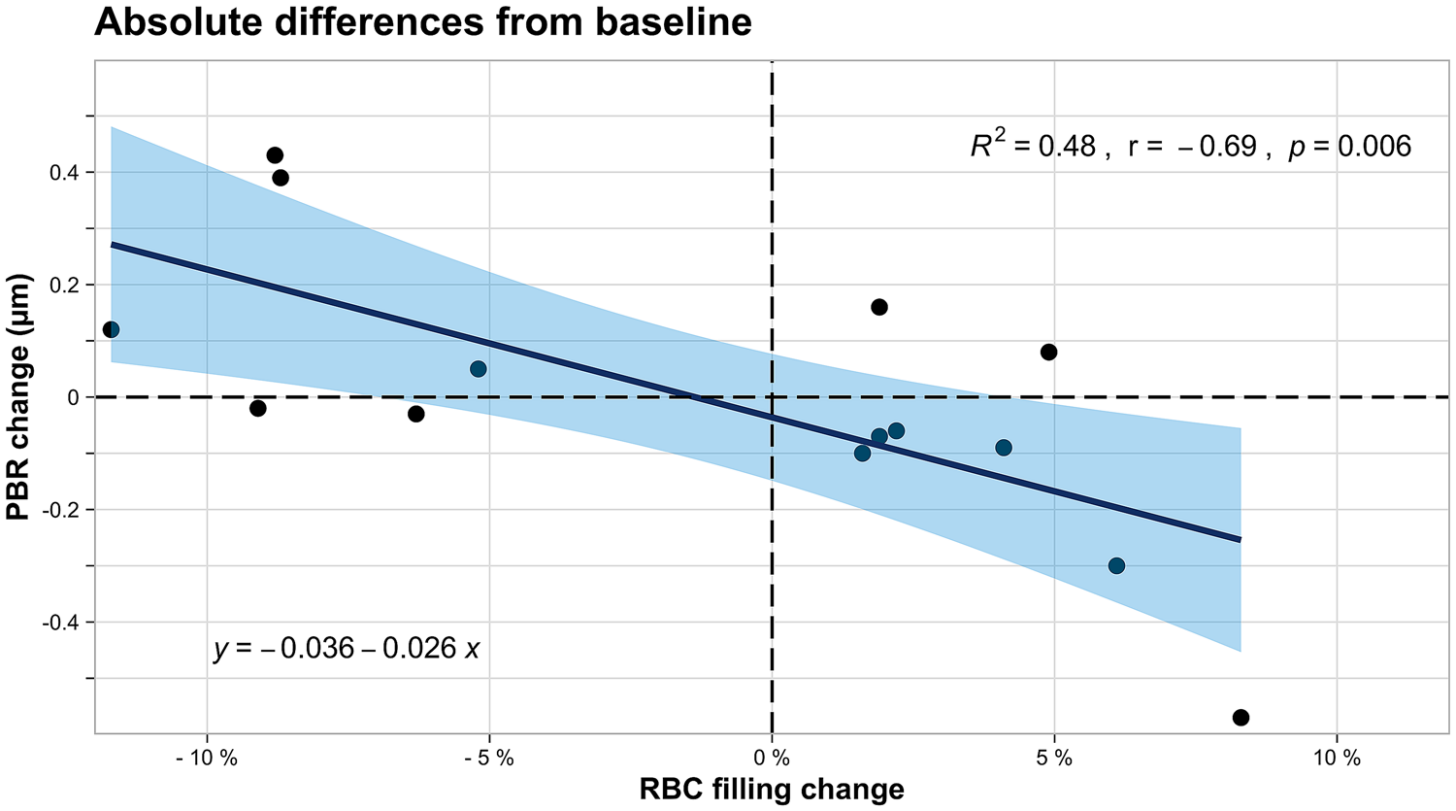
Scatterplot of change of PBR (%) and change of RBC filling (%). Linear regression lines overlaid, coefficients of determination, Spearman’s correlation coefficients and p-values are given

PBR and RBC filling percentage at baseline, PBR and RBC filling percentage at post-exercise (Fig. 2) as well as change of PBR and change of RBC filling percentage showed a strong negative correlation, respectively (Fig. 3). Linear regression analysis revealed a distinct association between change of PBR and change of RBC filling percentage (regression coefficient β: −0.026; 95% confidence interval: − 0.043 to − 0.009; p = 0.006).

## Discussion

We were not able to detect an immediate effect on ESL dimensions following aerobic exercise in this study. There was a negative association of PBR and RBC filling percentage at baseline, as has been described in previous studies [9, 15, 16]. This negative association of PBR and RBC filling percentage was maintained and even more pronounced after aerobic exercise, resulting also in a distinct association between change of PBR and change of RBC filling percentage. This aerobic exercise-stimulated association between the decrease of PBR and an increase of RBC filling percentage suggests that the endothelium and its ESL facilitate beneficial conditions for efficient perfusion in a hyperdynamic state like aerobic exercise.

In a previously published study, healthy volunteers performed an all-out running high-intensity interval training with one weekly session for four weeks [19]. Maximal exercise performance capacity (as measured by an incremental continuous running test) and PBR showed a negative correlation at baseline. The change in exercise performance capacity and the change in PBR showed an intensified negative correlation after the intervention indicating greater ESL thickness with improved exercise performance capacity [19]. It is intriguing to speculate that repeated endurance or high-intensity interval training might have a protective effect on the microvasculature as we did not find an immediate effect of a single aerobic-exercise on ESL dimension in our study. The underlying mechanisms have yet to be elucidated. A possible mechanism being discussed is an enhanced resistance of the ESL following repeated training sessions. For instance, moderate-intensity endurance training four times a week for 20 weeks performed by untrained young men lead to reduced syndecan-1 and heparan sulphate levels at rest and lower levels of these ESL degradation parameters after an incremental cycling test until exhaustion following the intervention [20]. Interestingly, maximal incremental cycling until exhaustion in 21 untrained men (mean age: 23 years) did not affect syndecan-1 and heparan sulphate levels [21] as opposed to an acute bicycle test with 45 min at an intensity of 70% of individual maximum oxygen uptake performed in individuals with a mean age of 50 yrs that led to an increase in levels of syndecan-1 and syndecan-4 [22]. Aging appears to be a factor contributing to ESL integrity. Lee et al. conclude that increased blood flow causes ESL degradation [22]. As we did not measure ESL shedding parameters, we do not know whether the preserved ESL dimensions were accompanied by stable levels of ESL shedding parameters or not. In addition, we did not assess maximum performance capacity before and after an intervention but were interested in the effect of a onetime aerobic-exercise on PBR. Hence, we cannot offer data regarding the association of individual performance capacity and PBR. Running time and PBR did not show a significant relation (data not shown). Neither valid nor total capillary density, nor the ratio of valid to total capillary density did change post-exercise. Hence, immediate capillary recruitment after a single aerobic-exercise was not detectable in our study but might be present when exercise is performed regularly or when measured in athletes. Patients with chronic heart failure have a lower valid and total capillary density, but a higher ratio of valid to total capillary density [23]. It suggests perfused capillary recruitment in an otherwise rarefied microcirculation. This increased ratio is also seen with age, body-mass-index, total cholesterol, and Framingham risk score [24] and may indicate a chronic adaptation process.

This was the first field study to assess the effect of aerobic exercise on ESL dimensions. ESL reactions of the sublingual mucosa may differ from tissues with high oxygen demand as heart and skeletal muscle in such scenarios. Participants older of age, concomitant arterial hypertension, dyslipidaemia or dysglycaemia may show other endothelial reactivity and tissue perfusion compared to the healthy volunteers we studied. As we did not conduct invasive measurements, we were not able to assess lactate levels.

We report the feasibility of an alternative scenario for intravital microscopy with automised analyses other than an in-hospital or a laboratory setting. We conclude that the endothelium of the microvasculature facilitates efficient perfusion in vessels reacting with an increased endothelial surface layer.

## Abbreviations

ESL: Endothelial surface layer
PBR: Perfused boundary region
RBC(s): Red blood cell(s)
